# Editorial for the Special Issue on Micro/Nano Devices for Blood Analysis

**DOI:** 10.3390/mi10100708

**Published:** 2019-10-18

**Authors:** Susana O. Catarino, Graça Minas, Rui Lima

**Affiliations:** 1 Center for MicroElectromechanical Systems (CMEMS-UMinho), University of Minho, Campus de Azurém, 4800-058 Guimarães, Portugal; 2 CEFT, Faculdade de Engenharia da Universidade do Porto (FEUP), Rua Roberto Frias, 4200-465 Porto, Portugal; 3 MEtRICs, Mechanical Engineering Department, University of Minho, Campus de Azurém, 4800-058 Guimarães, Portugal

The development of microdevices for blood analysis is an interdisciplinary subject that demands an integration of several research fields such as biotechnology, medicine, chemistry, informatics, optics, electronics, mechanics, and micro/nanotechnologies.

Over the last few decades, there has been a notably fast development in the miniaturization of mechanical microdevices, later known as microelectromechanical systems (MEMS), which combine electrical and mechanical components at a microscale level. The integration of microflow and optical components in MEMS microdevices, as well as the development of micropumps and microvalves, have promoted the interest of several research fields dealing with fluid flow and transport phenomena happening at microscale devices. 

Microfluidic systems have many advantages over macroscale by offering the ability to work with small sample volumes, providing good manipulation and control of samples, decreasing reaction times and allowing parallel operations in one single step. Despite the enormous scientific achievements that microfluidics have had in the last decades in the field of biomedical applications, this technology is still considered in an early stage, with some pullbacks, such as the difficulty to achieve a cost-effective large-scale production, and a lack of complete understanding of the physics of fluids at the microscale level over the biological species. Consequently, enormous efforts have been performed in microfabrication and microfluidics research to enhance the potential of microdevices to develop portable and point-of-care diagnostic devices, particularly for blood analysis. 

In this special issue, Catarino et al. [1] present one review paper on an overview of the techniques used for sorting and separation of red blood cells (RBCs) and the respective micro- and nanofabrication techniques, as well as examples of lab-on-a-chip devices with high potential for the integration of separation and detection tools in a single microfluidic platform [1]. 

Additionally, the special issue also contains 10 research papers covering different subjects related to microfluidic structures and cells characterization. Particularly, Kawaguchi et al. [2] have proposed a method to assess the changes in the rheological properties of a suspension containing fluorescent particles. On one side, the authors used a microchannel with a circular cross section and measured the distribution of suspended microparticles in the radial direction from the recorded images. On the other side, the authors evaluated the non-Newtonian rheological properties of the suspension using the velocity distribution obtained by the particle tracking velocimetry (PTV) and a power-law fluid model [2]. 

Three of the research papers focus are focused on assessing the deformability of RBCs in microchannels [3,4,5]. Takeishi et al. [3] numerically investigated the dynamics of RBCs flowing at different velocities in a narrow rectangular microchannel, for different capillary (Ca) numbers. The authors found that RBCs confined in the microchannel assumed a nearly unchanged biconcave shape at low Ca numbers, which became an asymmetrical slipper shape at moderate Ca numbers, and a symmetrical parachute shape at high Ca values, showing that the measurement of the configurations of the flowing cells can be a valuable tool to quantify the cell state [3]. 

Faustino et al. [4] have presented a passive microfluidic tool that provides the assessment of motions and deformations of RBCs of end-stage kidney disease (ESKD) patients (comparing patients with and without diabetes type II). The experimental flow studies were performed within a hyperbolic converging microchannel where single-cell deformability was assessed under a controlled homogeneous extensional flow field, using a high-speed video microscopy system. The velocities and deformability ratios were calculated for 27 individuals, 20 of them having ESKD, and the results showed that the proposed device was able to detect changes in the deformability ratio of the RBCs, allowing for distinguishing the samples from the healthy controls and the patients. The RBCs deformability of ESKD patients with and without diabetes was lower than of the healthy controls, with this difference being more evident for the group of ESKD patients with diabetes [4]. 

Vilas Boas et al. [5] reported an experimental study of the deformability and velocity assessment of healthy and artificially impaired red blood cells (RBCs), in narrow (8 µm width) polydimethylsiloxane (PDMS) microchannels, with the purpose of potentially mimicking malaria effects. The authors modified the RBCs by adding different concentrations of glucose, glutaraldehyde, or diamide in order to increase the cells’ rigidity, and obtained a velocity/deformability relation in the microchannel contraction, which shows great potential to relate the RBCs’ behavior with the various stages of malaria, helping to establish the development of new diagnostic systems towards point-of-care devices [5].

Kang and Kim [6] studied the RBCs aggregation and sedimentation rate in a microfluidic device, fabricated by xurography. In this study, multiple and periodic measurements were obtained by pulling blood from a pipette tip into parallel microfluidic channels, and quantifying the image intensity. The authors have considered two indices (aggregation index and erythrocyte-sedimentation-rate aggregation index) and have evaluated the effect of hematocrit and dextran solution on these values, showing that the erythrocyte-sedimentation-rate aggregation index varies linearly within a specific concentration of dextran solution [6].

Ponmozhi et al. [7] developed and characterized a low-cost microfluidic device for adhesion tests in polymeric surfaces, which can be fabricated in a laboratory with low resources. The fabrication method consisted of a modification of the existing PDMS soft lithography method and, therefore, is compatible with sealing methods and equipment of most microfluidic laboratories. The molds were produced by xurography, and the fabrication method was tested by evaluating the bacterial adhesion in five different materials, with different surface hydrophobicity and charges. The authors also performed a computation fluid dynamics analysis of the flow in the microfluidic device [7].

Four research papers studied clinical applications of microfluidic devices, in particular for quantification of tumor markers, as well as the characterization of the morphological properties of other cell cultures and counting cells. In particular, Sugita et al. [8] developed a method to efficiently obtain in vitro multinucleated cells and characterized their morphological properties. The authors seeded a Xenopus tadpole epithelium tissue-derived cell line (XTC-YF) in different hydrophobicity dishes and with or without supplements, and verified that 88% of the cells cultured on a less hydrophilic dish in medium supplemented with Y-27632 became multinucleate 48 h after seeding [8]. 

Since metastatic cancer cells are known to have a smaller cell stiffness than healthy cells, Nakamura et al. [9] developed a simple microfluidic system to assess metastatic capacity of the cancer cells from a mechanical point of view, by evaluating the viscoelastic properties of cancer cells on a tapered microchannel. Two metastasis B16 melanoma variants (B16-F1 and B16-F10) were examined and the shape recovery process of the cell from a compressed state was evaluated with the Kelvin–Voigt model. The shape recovery time constant became larger as cancer cells had higher metastatic potential [9].

Gao et al. [10] developed a high-throughput centrifugal microfluidic device for detecting carcinoembryonic antigen (CEA) in serum, which is a broad-spectrum tumor marker used in clinical applications, without the need for cumbersome washing steps normally used in immunoreactions. This centrifugal microdevice contains 14 identical pencil-like units, and the CEA molecules were separated from the bulk serum for subsequent immunofluorescence detection using density gradient centrifugation in each unit simultaneously. The proposed device can achieve a high-throughput detection [10].

Finally, Fang et al. [11] presented a low cost and small size flow cytometer on a microfluidic chip, integrating an inline lens-free holographic microscope. The authors proposed an S-type microchannel with a pulse injection flow, and obtained a less than 2% cell counting error. This on-chip flow cytometer can continuously count cells and continuously collect a large number of cell images for subsequent cell analysis, and is in full compliance with the current development trend of point-of-care testing [11].

We hope this issue can provide an opportunity to the engineering and biomedical community, and those who are interested in the general field of MEMS and micro/nanofluidics, to access novel knowledge and information, especially in its applications to biomedical areas. Particularly, we hope this issue can contribute as a display of the latest achievements, breakthroughs, challenges and future trends in microdevices for diagnostics and blood analysis, micro- and nanofluidics, technologies for flows visualization, MEMS, biochips and lab-on-a-chip devices and their application to research and industry. 

Finally, we would like to congratulate, acknowledge and thank all the authors for submitting their original manuscripts to this special issue, as well as all the reviewers for the time and help to improve the quality of the submitted papers.
